# Ba_3_Mg_3_(BO_3_)_3_F_3_ polymorphs with reversible phase transition and high performances as ultraviolet nonlinear optical materials

**DOI:** 10.1038/s41467-018-05575-w

**Published:** 2018-08-06

**Authors:** Miriding Mutailipu, Min Zhang, Hongping Wu, Zhihua Yang, Yihan Shen, Junliang Sun, Shilie Pan

**Affiliations:** 1 CAS Key Laboratory of Functional Materials and Devices for Special Environments, Xinjiang Technical Institute of Physics & Chemistry, CAS, Xinjiang Key Laboratory of Electronic Information Materials and Devices, 40-1 South Beijing Road, Urumqi, 830011 China; 2 University of Chinese Academy of Sciences, Beijing, 100049 China; 3 College of Chemistry and Molecular Engineering, Peking University, Beijing, 100871 China

## Abstract

Nonlinear optical (NLO) materials are the vital components of future photoelectric technologies as they can broaden the tunable wavelength range supplied by common laser sources. However, the necessary prerequisites for a practical NLO material are rather strict. Accordingly, considerable efforts have been focused on finding potential NLO materials. Here we report two asymmetric beryllium-free borates *Pna*2_1_- and *P*$\bar 6$2*m*-Ba_3_Mg_3_(BO_3_)_3_F_3_ featuring NLO-favorable ^2^_∞_[Mg_3_O_2_F_3_(BO_3_)_2_] layered structures. The reversible phase transition among two polymorphs was demonstrated by multiple experimental tests. The optical measurements reveal that *Pna*2_1_-Ba_3_Mg_3_(BO_3_)_3_F_3_ possesses the optical properties required for ultraviolet NLO applications. Remarkably, *Pna*2_1_-Ba_3_Mg_3_(BO_3_)_3_F_3_ has a large laser damage threshold, a deep-ultraviolet cutoff edge, a favorable anisotropic thermal expansion as well as the capacity of insolubility in water. These optical properties can be comparable or superior to that of commercial NLO material *β*-BaB_2_O_4_, which make *Pna*2_1_-Ba_3_Mg_3_(BO_3_)_3_F_3_ a promising ultraviolet NLO material.

## Abstract (web summary)

Nonlinear optical crystals suitable for the UV spectral region could simplify short-wavelength generation and make it more efficient. Here, the authors design and demonstrate that one of two asymmetric borate polymorphs exhibits promising optical and mechanical properties for generating UV light.

## Introduction

Nonlinear optical (NLO) materials applied from ultraviolet (UV) to infrared spectral ranges are the vital components of future photoelectric technologies^1–4^. Accordingly, considerable and sustained efforts have been focused on designing and synthesizing new potential NLO materials^5–9^. Generally speaking, a practical UV NLO material should simultaneously satisfy the following criteria: a noncentrosymmetric structure; a broad transparent spectral range down to the UV region (*λ* < 400 nm); a relatively large second-order nonlinear coefficient (*d*_*ij*_ > 0.39 pm/V); a moderate birefringence to satisfy the phase-matching condition, chemical stability (non-deliquescent), and resistance to laser (large laser damage threshold, large LDT); and ease of growth of large single crystals with high optical quality^10^. Targeting above conditions, until now, the borate system plays a dominant role in UV NLO materials because borates possess wide optical transparency windows, varied acentric structure types, high LDTs, and large polarizabilities^11–18^. Therefore, a variety of commercial borate-based UV NLO materials have been widely investigated and developed. Among them, borates with planar [BO_3_]^3−^ anionic groups have always been at the center of the search for NLO materials with large second-harmonic generation (SHG) response and birefringence, which mainly benefits from the *π*-conjugated molecular orbitals of [BO_3_]^3−^ units^19–22^. Based on this, several borates with [BO_3_]^3−^ NLO-active units in coplanar and aligned configuration were continuously reported with good optical properties, including KBe_2_BO_3_F_2_ (KBBF)^19^, BaAlBO_3_F_2_^20^, NaSr_3_Be_3_B_3_O_9_F_4_^21^, Li Sr(BO)^22^, etc.

For the sake of finding new novel UV or even deep-UV (*λ* < 200 nm) NLO crystals, an effective design tactic is to introduce fluorine into the structural framing of borate system based on the following considerations. First, the fluorine with the largest electronegativity (*χ* = 3.98) can widen the transparency area and make the cutoff edge of borates blue-shift into UV or even deep-UV spectral region^19–21,23–25^. Second, substituting oxygen with fluorine in [BO_4_]^5−^ maternal blocks to form [BO_*x*_F_4−*x*_]^(*x*+1)−^ (*x* = 0, 1, 2, 3) fundamental building blocks can make the anionic groups more flexible, and the negative effective charges at the terminal oxygen will be partly reduced, resulting in a suitable SHG coefficient and birefringence^26–29^. Third, the introduction of fluoride-containing flux can effectively decrease the viscosity of the borate systems during the growth of single crystals, which is a desirable choose for growing the large single crystals^20,24,25,30^.

For us, besides selecting the appropriate system (borate) and introducing anion (fluorine), how to effectively design UV NLO materials with presupposed structures and properties is also critically important. The following ideas were considered for designing the target molecules: first, in order to manipulate the B–O fundamental building blocks into isolated planar [BO_3_]^3−^ configuration, we adjusted the ratio of cations and boron to >1.0 based on the rules proposed by Becker^31^. Second, we use the strategy of chemical cosubstitution to make structural modifications based on the classic NLO materials with the goal of obtaining new molecular structures contain NLO-favorable layered structures^32,33^.

Motivated by these, considering that *s*-block elements Be and Mg, Sr and Ba appear tightly clustered in the periodic table with the similar electronic configurations, sharing many common physicochemical properties. Therefore, we attempted to substitute the Be and Sr atoms of Sr_2_Be_2_B_2_O_7_ (SBBO)^1^ with Mg and Ba atoms through chemical cosubstitution idea to create new SBBO-like borates, which leads us to discover two new beryllium-free borates, *Pna*2_1_- and *P*$\bar 6$2*m*-Ba_3_Mg_3_(BO_3_)_3_F_3_ (*Pna*2_1_- and *P*$\bar 6$2*m*-BMBF), featuring SBBO-like layered structures without structural instability. The ^2^_∞_[Mg_3_O_2_F_3_(BO_3_)_2_] layers in both of them not only preserve the NLO-favorable structural habits of SBBO, but also introduce the Mg–F bonds served between layers as bridges to enhance the interlayer interaction. Based on the top designing molecule structures, large-size single crystals of *Pna*2_1_-BMBF were also grown through finding the suitable flux system. The optical measurements based on the crystal reveal that *Pna*2_1_-BMBF possesses the NLO properties required for the UV NLO applications, which indicates that *Pna*2_1_-BMBF is a potential UV NLO material.

## Results

### The reversible phase transition between two polymorphs

**Fig. 1:**
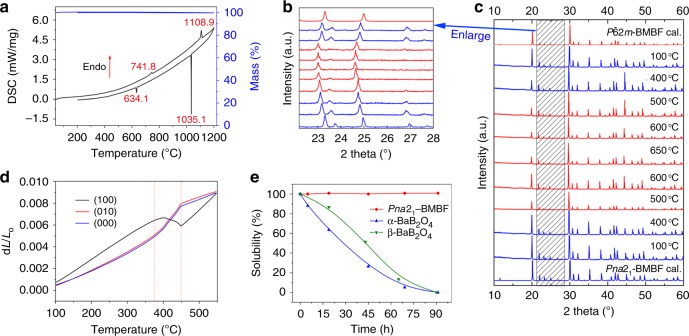
Basic physicochemical properties of Ba_3_Mg_3_(BO_3_)_3_F_3_. **a** Thermal gravimetric and differential scanning calorimetry (DSC) curves. Endo means the endothermic direction. There are two remarkable endothermic peaks (741.8 and 1108.9 °C) on the heating curve and two exothermic peaks (634.1 and 1035.1 °C) on the cooling curve, respectively. **b**, **c** High-temperature in situ powder X-ray diffraction data circularly collected in the region of 100–650 °C. The experimental and caculated powder X-ray diffraction patterns of *Pna*2_1_-Ba_3_Mg_3_(BO_3_)_3_F_3_ (blue lines) and *P*$\bar 6$2*m*-Ba_3_Mg_3_(BO_3_)_3_F_3_ (red lines). The enlarged version of **c** in the 2*θ* region from 22.3 to 28.2° is shown in **b** to better understand the phase transition behavior between two polymorphs. **d** Thermal expansion measurements tested by (100), (010), and (001) *Pna*2_1_-Ba_3_Mg_3_(BO_3_)_3_F_3_ crystal plates. d*L* and *L*_0_ mean the change in crystal plates length and initial length of crystal plates, respectively. **e** Solubility experiments of *Pna*2_1_-Ba_3_Mg_3_(BO_3_)_3_F_3_, *α-* and *β*-BaB_2_O_4_

Single crystal X-ray diffraction reveals that two new borates crystallize into the asymmetric space group of *Pna*2_1_ (No. 33) and *P*$\bar 6$2*m* (No. 189), respectively (Table 1 and Supplementary Table 1). Interestingly, the molecular formula of both phases was determined as Ba_3_Mg_3_(BO_3_)_3_F_3_ through structure solutions, which have the same simplest molecular formula with BaMgBO_3_F (monoclinic, *Cc*) reported by Li. et al.^34^. And the relatively low temperature factors and residual factors verify the correctness of those two crystal models, which also indicates the existence of two polymorphs. Furthermore, the BMBF polymorphs and phase transitions are further confirmed by high-temperature in situ powder X-ray diffraction and thermal expansion coefficients experiment: based on the results of thermal gravimetric and differential scanning calorimetry analysis (Fig. 1a), we make circular heating and cooling the *Pna*2_1_-BMBF samples in the region of 100–650 °C. When the temperature rises to 500 °C, the *Pna*2_1_-BMBF phase start to transfer into *P*$\bar 6$2*m-*BMBF phase, and *P*$\bar 6$2*m-*BMBF is stable up to 650 °C. When the temperature begins to decrease, *P*$\bar 6$2*m-*BMBF phase can also transfer into *Pna*2_1_-BMBF, indicating the reversibility of this phase transition behavior. More intuitively, the disappearance and appearance of X-ray diffraction peaks at about 2*θ* = 23.690, 26.994, and 27.686° in BMBF series can help us to judge this phase transition (Fig. 1b, c). The measured average thermal expansion coefficients based on the three as-polished (100), (010), and (001) wafers occur sudden change at about 450 °C, which is caused by the phase transitions of BMBF series (Fig. 1d).

**Table 1:** Crystallographic data for *Pna*2_1_-and *P*$\bar 6$2*m*-Ba_3_Mg_3_(BO_3_)_3_F_3_

Empirical formula	Ba_3_Mg_3_(BO_3_)_3_F_3_	Ba_3_Mg_3_(BO_3_)_3_F_3_
Formula weight	718.38	718.38
Crystal system	Orthorhombic	Hexagonal
Space group	*Pna*2_1_ (No. 33)	*P*$\bar 6$2*m* (No. 189)
*a* (Å)	8.0740(3)	8.804(3)
*b* (Å)	15.3072(7)	8.804(3)
*c* (Å)	8.8218(4)	4.025(3)
*Z*	4	1
Volume (Å^3^)	1090.29(8)	270.2(2)
Reflns collected/unique	11,605/2377 [*R*(int) = 0.0243]	1695/261 [*R*(int) = 0.0386]
Completeness (%)	99.8	100
Goodness of fit on *F*^2^	1.233	1.251
Final *R* indices [*F*_o_^2^ > 2*σ*(*F*_o_^2^)]^a^	*R*_1_ = 0.0118, *wR*_2_ = 0.0249	*R*_1_ = 0.0204, *wR*_2_ = 0.0394
*R* indices (all data)^a^	*R*_1_ = 0.0120, *wR*_2_ = 0.0250	*R*_1_ = 0.0207, *wR*_2_ = 0.0395

*R* means residual factor

^a^*R*_1_ = Σ||*F*_o_| − |*F*_c_||/Σ|*F*_o_| and *wR*_2_ = [Σ*w*(*F*_o_^2^ − *F*_c_^2^)^2^/ Σ*wF*_o_^4^]^1/2^ for *F*_o_^2^ > 2*σ*(*F*_o_^2^)

### Crystal structures of *Pna*2_1_- and *P*$\bar 6$2*m*-BMBF

**Fig. 2:**
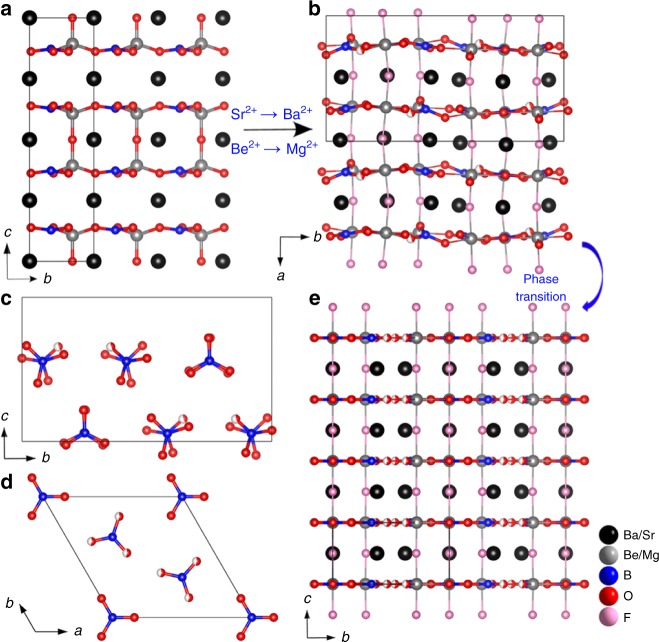
Crystal structural features of *Pna*2_1_-Ba_3_Mg_3_(BO_3_)_3_F_3_. **a**, **b** The structural evolution from Sr_2_Be_2_B_2_O_7_ to *Pna*2_1_-Ba_3_Mg_3_(BO_3_)_3_F_3_. **c**, **d** The orientation of [BO_3_]^3−^ groups in *Pna*2_1_- and *P*$\bar 6$2*m-*Ba_3_Mg_3_(BO_3_)_3_F_3_, respectively. **e** The layered structure of derivative *P*$\bar 6$2*m-*Ba_3_Mg_3_(BO_3_)_3_F_3_ derived by phase transition from *Pna*2_1_-Ba_3_Mg_3_(BO_3_)_3_F_3_

The structural evolution among SBBO, *Pna*2_1_-, and *P*$\bar 6$2*m-*BMBF are shown in Fig. 2, which verifies the validity of structure-oriented design strategy. As shown in Fig. 2a, b, e, BMBF series possess a similar layered crystal structures, their crystal structures are both composed of ^2^_∞_[Mg_3_O_2_F_3_(BO_3_)_2_] layers (Supplementary Figure 1) along the *a*-axis for *Pna*2_1_-BMBF and *c*-axis for *P*$\bar 6$2*m*-BMBF, and those single layers are further connected by the Mg–F bonds to construct a three-dimensional framework with tunnels running along the *c* (or *a*)-axis. In the asymmetric unit of *Pna*2_1_-BMBF, the Ba, Mg, B, and F atoms occupy three crystallographically unique positions, for the O atoms there are nine unique positions. While for *P*$\bar 6$2*m*-BMBF with higher symmetry, the Ba, Mg, B, O, and F atoms occupy one, one, two, two, and one unique positions (Supplementary Tables 2, 3, 5, and 6). In both BMBF polymorphs, the B atoms possess only one coordination type, the [BO_3_]^3−^ triangles (Fig. 2c, d). The B–O bond distances and O–B–O bond angles locate in the range from 1.341 to 1.395 Å, 117.6 to 120.9 ° for *Pna*2_1_-BMBF, and 1.349 to 1.381 Å, 117.0 to 122.8 ° for *P*$\bar 6$2*m*-BMBF, respectively (Supplementary Tables 4 and 7). The Mg atoms in both polymorphs are six-coordinated into the MgO_4_F_2_ octahedra with four O and two F atoms locating in the equatorial and axial positions, respectively. Three MgO_4_F_2_ units share three O atoms to generate a six-membered ring cluster Mg_3_O_9_F_6_ (Supplementary Figure 2), then those clusters link together via the axial Mg–F bonds to build an isolated ^3^_∞_[Mg_3_O_9_F_3_] triangular prism (Supplementary Figure 3). Interestingly, two [BO_3_]^3−^ units locate in and out of the six-membered ring Mg_3_O_9_F_6_ to form polymer and further polymerize into ^2^_∞_[Mg_3_O_2_F_3_(BO_3_)_2_] infinite layers, and the connection is by bridging Mg–F bonds to form a three-dimensional multilayered structure. All the Ba atoms are coordinated into BaO_*n*_F_*m*_ (*m* + *n* = 10 and 11) polyhedra, located between the adjacent ^2^_∞_[Mg_3_O_2_F_3_(BO_3_)_2_] layers, which is quite like the arrangement of the Sr atoms in SBBO^1^.

Structurally, the triangular [BO_3_]^3−^ units in both *Pna*2_1_- and *P*$\bar 6$2*m*-BMBF adopt nearly coplanar configuration. This is propitious to generate large SHG response and birefringence because of the strong anisotropy resulting from their different polarizabilities in different directions, which is similar to the NLO materials containing *π*-planar triangular [CO_3_]^2−^^35,36^. The F–Mg–F bonds act as bridge-linking role to connect the neighboring layers, which is similar to the [B_2_O_5_]^4−^ dimers in LiNa_5_Be_12_B_12_O_33_^37^ and the [B_3_O_6_]^3−^ groups in Cs_3_Zn_6_B_9_O_21_^38,39^. Further, the electrostatics force of the interaction between layers in *Pna*2_1_- and *P*$\bar 6$2*m*-BMBF was evaluated and |*F*_Mg–F_| is stronger than that of |*F*_K–F_| in KBBF (Supplementary Table 8), indicating that the electrostatic interactions between Mg–F bonds in *Pna*2_1_- and *P*$\bar 6$2*m*-BMBF provide better linkage of neighboring layers, which is favorable to improve the layering tendency and the same results can be concluded from the ease of the growth of block-like crystals of BMBF series than that of KBBF.

### Crystal growth and optical quality of *Pna*2_1_-BMBF

**Fig. 3:**
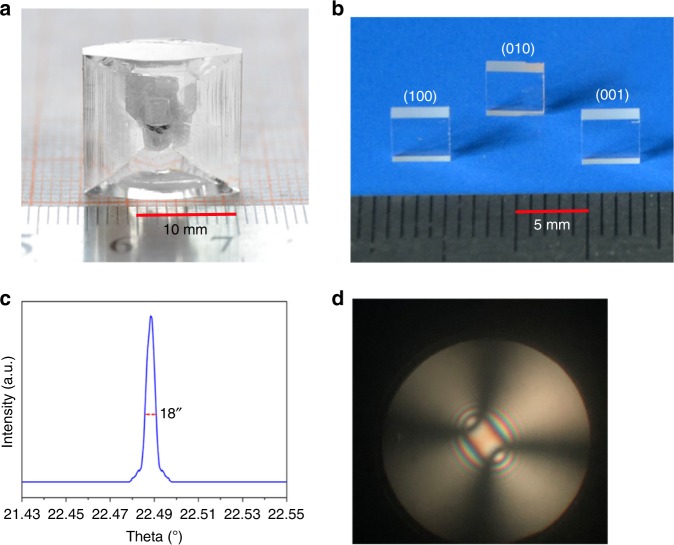
The as-grown single crystal and optical quality experiments of *Pna*2_1_-Ba_3_Mg_3_(BO_3_)_3_F_3_. **a** Photograph of the as-grown crystal with dimensions up to 16 × 14 × 8 mm^3^ grown by the top-seeded solution growth method. **b** The cut and polished (100), (010), and (001) wafers with dimensions up to 4 × 4 × 1 mm^3^. The crystals were cut plane parallel and polished to optical quality using a Unipol-300 grinding/polishing machine. **c** The high-resolution X-ray diffraction rocking curve for (100) wafer. The full-width at half-maximum on (100) face of the *Pna*2_1_-Ba_3_Mg_3_(BO_3_)_3_F_3_ crystal is about 18″, indicating high crystalline quality. **d** The conoscopic interference pattern of *Pna*2_1_-Ba_3_Mg_3_(BO_3_)_3_F_3_ crystal. All the images and elements of them were created by ourselves

After various attempts, *Pna*2_1_-BMBF crystal with dimensions up to 16 × 14 × 8 mm^3^ has been grown at the optimal conditions (Fig. 3a). *Pna*2_1_-BMBF crystal has never cracked during cutting and polishing into (100), (010), and (001) wafers (Fig. 3b). The crystallization quality of the as-grown crystal was checked by the X-ray rocking curve. As shown in Fig. 3c, the full-width at half-maximum on (100) face of *Pna*2_1_-BMBF crystal was measured to be 18″, indicating high crystalline quality. The conoscopic interference pattern (Fig. 3d) shows that the as-grown *Pna*2_1_-BMBF crystal is biaxial, optical homogeneous, and does not have any strain inside. When taken together, *Pna*2_1_-BMBF crystal shows high optical quality, and the optical measurements based on this crystal are reliable.

### High performances as UV NLO materials

*Pna*2_1_-BMBF was subjected to physicochemical property characterizations based on the single-crystal level.

Thermal expansion coefficients are vital parameters for crystal growth and application in devices. As can be seen in Fig. 1d, the average thermal expansion coefficients in the region from 100 to 450 °C are evaluated to be *α*_a_ = *α*(100) = 2.17 × 10^−5^ K^−1^, *α*_b_ = *α*(010) = 1.63 × 10^−5^ K^−1^, and *α*_c_ = *α*(001) = 1.53 × 10^−5^ K^−1^, respectively. The value of *α*_a_/*α*_c_ is about 1.43 for *Pna*2_1_-BMBF crystal, which is smaller than that of *β*-BaB_2_O_4_ (*β*-BBO) (*α*_c_/*α*_a_ = 9)^40^. Therefore, *Pna*2_1_-BMBF crystal exhibits a more favorable anisotropic thermal expansion, which will effectively protect the crystals from cracking caused by thermal expansion during crystal growth and optical devices fabrication.

To study the water solubility of *Pna*2_1_-BMBF, we dipped the target *Pna*2_1_-BMBF (0.113 g) crystal as well as the references *α*- and *β*-BBO crystals (0.159 and 0.105 g) into 20 mL de-ionized water at room temperature. As plotted in Fig. 1e, the weight of *Pna*2_1_-BMBF did not change in the measured time ranges, while for *α*- and *β*-BBO crystals, they were dissolved in water in 90 h. These indicate that *Pna*2_1_-BMBF is chemically stable and not soluble in the water.

We carried out the SHG measurements by the Kurtz–Perry method^41^ with incident lasers at *λ* *=* 1064 nm. Meanwhile, the well-known NLO material KH_2_PO_4_ (KDP) was used as the reference. The comparison of SHG signals produced by *Pna*2_1_- and *P*$\bar 6$2*m-*BMBF polycrystalline samples in the same particle sizes ranging from 200 to 250 μm reveals that *Pna*2_1_- and *P*$\bar 6$2*m-*BMBF exhibit suitable SHG responses of ∼1.8 and 2.0 × KDP, respectively. Such SHG responses are large enough for the applications of UV NLO materials and it is also comparable to those reported borates with deep-UV cutoff edges, such as KBBF (1.26 × KDP)^19^, LiNa_5_Be_12_B_12_O_33_^37^ (1.4 × KDP), and Li_4_Sr(BO_3_)_2_ (2 × KDP)^22^. According to the results, the SHG efficiency increases with the raising particle sizes, indicating that both BMBF polymorphs exhibit type I phase-matching behavior in 1064 nm incident lasers. In addition, the effective NLO coefficients (*d*_eff_) of *Pna*2_1_- and *P*$\bar 6$2*m-*BMBF crystal are estimated to be about 1.3 and 1.4 × KDP according to the square roots of the ratios of their corresponding SHG signal intensities. In order to verify the results, large sizes of single crystals should be grown and cut to evaluate the SHG coefficients based on the crystal level, which is discussed in detail in the following parts.

**Fig. 4:**
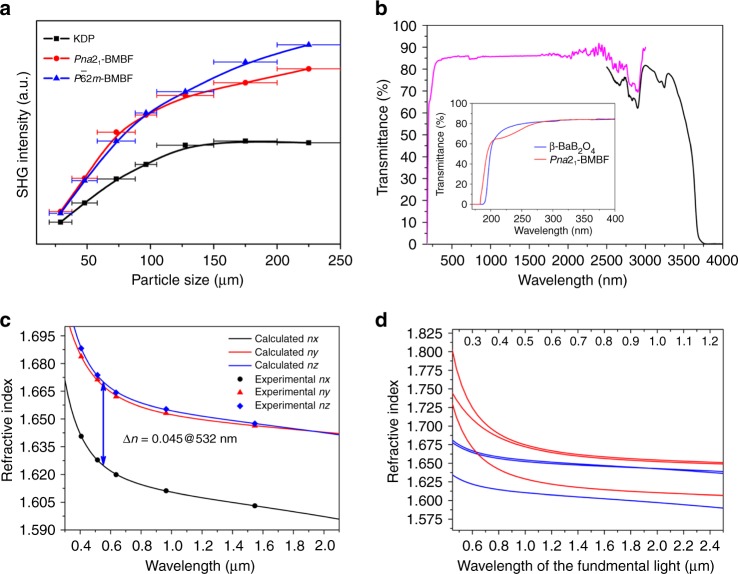
Optical properties of *Pna*2_1_-Ba_3_Mg_3_(BO_3_)_3_F_3_. **a** Powder second-harmonic generation data for Ba_3_Mg_3_(BO_3_)_3_F_3_ polymorphs at 1064 nm laser radiation. The well-known NLO material KH_2_PO_4_ (KDP) was used as the reference. The curves are drawn to guide the eyes, and are not fit to the data. The error bars from left to right correspond to sieved crystal particle size ranges: 20–38; 38–55; 55–88; 88–105; 105–150; 150–200; and 200–250 μm. **b** Transmission spectrum of *Pna*2_1_-Ba_3_Mg_3_(BO_3_)_3_F_3_ crystal. Transmittance curve (magenta line) between 180 and 2500 nm was collected by a Shimadzu Solid Spec-3700DUV Spectrophotometer. Transmittance curve (black line) between 2500 and 4000 nm was collected by a Shimadzu IRAffinity-1 spectrometer. Inset gives transmittance curves between 170 and 400 nm for *Pna*2_1_-Ba_3_Mg_3_(BO_3_)_3_F_3_ (red line) and *β*-BaB_2_O_4_ (blue line). The ultraviolet cutoff edge of *Pna*2_1_-Ba_3_Mg_3_(BO_3_)_3_F_3_ is about 184 nm, which is 5 nm shorter than that of *β*-BBO (189 nm). **c** The refractive index dispersion curves for the *Pna*2_1_-Ba_3_Mg_3_(BO_3_)_3_F_3_ crystal. **d** The refractive index dispersion curves for fundamental (blue lines) and the second-harmonic (red lines) light. Based on the Sellmeier equations, when considering the type I phase-matching condition, *n*(*ω*) = *n*(2*ω*), the shortest type I phase-matching wavelengths in the *xz* and *xy* plane are evaluated to be 310 and 322 nm

The as-grown crystals were cut and optically polished to 1 mm thickness for the spectrum measurement. As presented in Fig. 4b, the *Pna*2_1_-BMBF crystal exhibits a broad transmission of 184–3780 nm, indicating that the application of *Pna*2_1_-BMBF crystal can cover a spectral range from UV to near-infrared. Obviously, the UV cutoff edge of *Pna*2_1_-BMBF is about 184 nm, which is 5 nm shorter than that of *β*-BBO (189 nm) in the same measured condition. And also the high transmittance of *Pna*2_1_-BMBF in the UV spectral range makes it possible to be applied as a UV NLO material. The short cutoff edge is beneficial to obtain high LDT, therefore, LDT measurements were carried out using a pulsed nanosecond laser (1064 nm, 10 ns, and 10 Hz). A well-polished high-quality (001) wafer of *Pna*2_1_-BMBF has a LDT of ∼6.2 GW/cm^2^, which is comparable to *β*-BBO crystal (∼6.0 GW/cm^2^) at the same experimental conditions.

The refractive indices of *Pna*2_1_-BMBF were measured using prism coupling method. As it follows the inequality *n*_*z*_ − *n*_*y*_ < *n*_*y*_ − *n*_*x*_, which depicts that *Pna*2_1_-BMBF is a negative biaxial crystal (consistent with results of conoscopic interference patterns), with the birefringence (Δ*n* = *n*_*z*_ − *n*_*x*_) ranging from 0.044@1064 nm to 0.045@532 nm and the values are comparable with those of the commercial UV NLO materials LiB_3_O_5_ and CsLiB_6_O_10_ (Δ*n* = 0.045 and 0.049@1064 nm)^10^. A least-square method was used to fit the dispersion parameters of the refractive indices, *n*_*i*_, using the Sellmeier Eq. (1):

1 $$n_i^2 = {{A}} + \frac{{{B}}}{{\lambda ^2 - {{C}}}} - {{D}}\lambda ^2$$

where *λ* represents the wavelength in units of µm, and *A*, *B*, *C*, and *D* are the Sellmeier parameters. The Sellmeier Eq. (1) deduced by the least-square fitting of all the measured refractive indices have been fitted as:

2 $$\begin{array}{ccccc}\\ n_x^2 = & 2.58654 + \frac{{0.01672}}{{\lambda ^2 - 0.00904}} - 0.00987\lambda ^2\\ \\ n_y^2 = & 2.71284 + \frac{{0.02264}}{{\lambda ^2 + 0.01829}} - 0.00486\lambda ^2\\ \\ n_z^2 = & 2.72604 + \frac{{0.01908}}{{\lambda ^2 - 0.01344}} - 0.00819\lambda ^2\\ \end{array}$$

The calculated and experimental values of the refractive indices at five different wavelengths are summarized in Supplementary Table 9. The calculated values are consistent with experimental ones to the fourth decimal place, which indicates that the fitted Sellmeier equations based on the current data are reliable (Fig. 4c). Based on above fitted Sellmeier equations, when considering the type I phase-matching condition, *n*(*ω*) = *n*(2*ω*), the shortest type I phase-matching wavelengths in the *xz* and *xy* plane are evaluated to be 310 and 322 nm (Fig. 4d), respectively. Therefore, the *Pna*2_1_-BMBF crystal can generate 532 and 355 nm light by direct second and third harmonic generation from a 1064 nm laser.

**Fig. 5:**
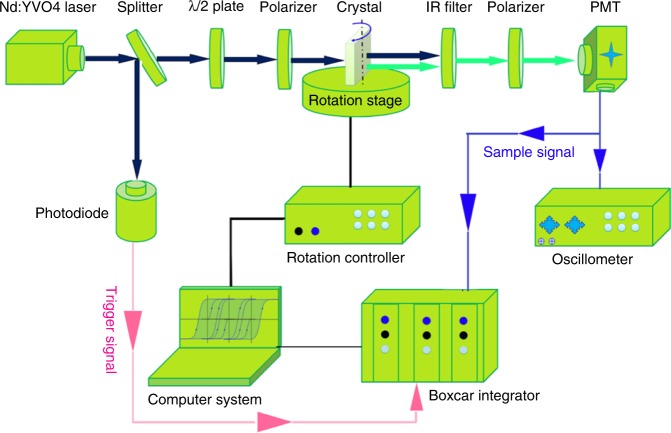
Schematic diagram of the Maker fringe experiment using an Nd:YVO_4_ laser. A Q-switched Nd:YVO_4_ laser system was adopted as fundamental light source with the following experimental conditions: fundamental wavelength: 1064 nm; repetition frequency: 10 kHz; and pulse width: 10 ns. The second-harmonic signal, which changed with rotation angle of *Pna*2_1_-Ba_3_Mg_3_(BO_3_)_3_F_3_ crystal, was detected by a photomultiplier tube, averaged by a fast-gated integrator and boxcar integrator, and then recorded by computer system. The *Pna*2_1_-Ba_3_Mg_3_(BO_3_)_3_F_3_ crystal is at the waist of the fundamental light with a beam diameter of 1.0 mm

**Fig. 6:**
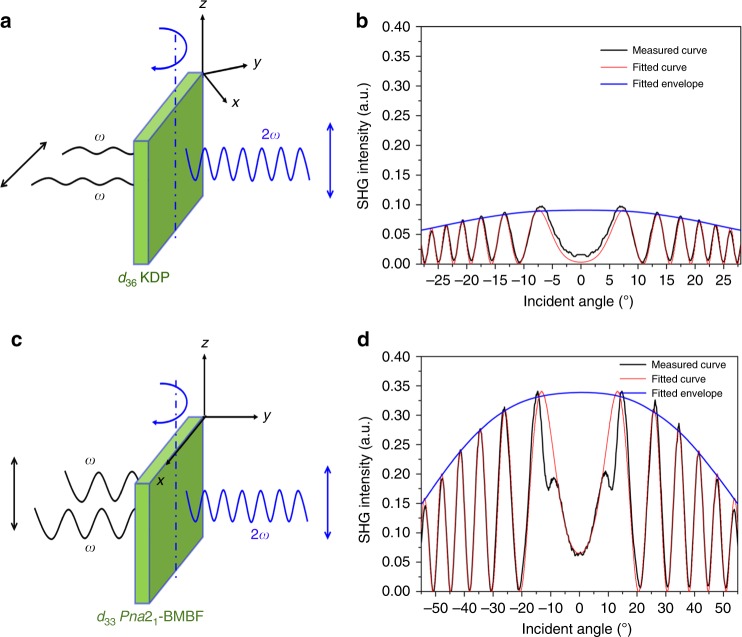
Maker fringe measurement for *Pna*2_1_-Ba_3_Mg_3_(BO_3_)_3_F_3_ crystal. **a**, **c** The orientations of as-grown crystal plates for measuring the NLO coefficients. **b**, **d** The measured and calculated Maker fringe data as well as fitted envelope for *d*_36_ of KH_2_PO_4_ (KDP) and *d*_33_ of *Pna*2_1_-Ba_3_Mg_3_(BO_3_)_3_F_3_. The data were fitted according to the Maker fringe theory and the detailed fitting procedure is available in the Methods section

In order to determine the individual NLO coefficients of *Pna*2_1_-BMBF, Maker fringe measurements were carried out. The schematic of the Maker fringe measurement system is shown in Fig. 5. For the SHG coefficient measurements, the orientation of the crystals and the relationships of *P*_*ω*_ and P_2*ω*_ are shown in Fig. 6a, c. The (001)-cut and (100)-cut plane-parallel uncoated plates of *Pna*2_1_-BMBF crystals (4 mm × 4 mm × 1 mm) were used to measure the related NLO coefficients. And also a (110)-cut plate (5 mm × 5 mm × 2 mm) of the KDP crystal was used to measure the *d*_36_ (KDP) NLO coefficient as the reference. By fitting the calculated Maker fringes based on the current measurements and calculations, the NLO coefficient of the *Pna*2_1_-BMBF crystal relative to *d*_36_ (KDP) has been determined as *d*_33_ = 1.3 × *d*_36_ (KDP) (Fig. 6b, d). However, the Maker fringe for *d*_31_ is too weak to be observed. As *d*_36_ coefficient of KDP is 0.39 pm/V, the absolute NLO coefficients of *Pna*2_1_-BMBF crystal based on the measurements and calculations are evaluated to be *d*_33_ = 0.51 pm/V.

## Discussion

**Fig. 7:**
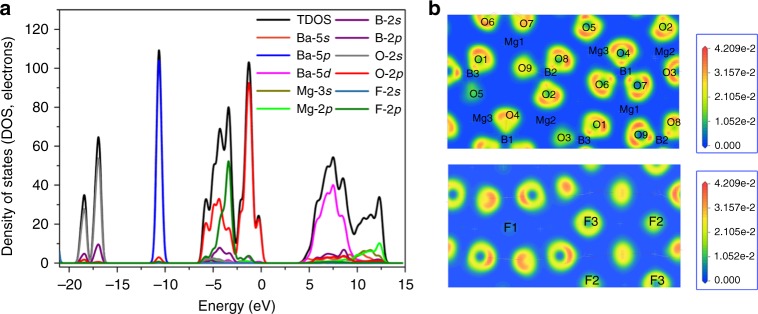
Theoretical calculation results of *Pna*2_1_-Ba_3_Mg_3_(BO_3_)_3_F_3_. **a** Partial and total density of states with the energy region from −22 to 15 eV. **b** The electron localization function diagrams slice along the *bc* plane (top of **b**) with the slice position of (5.70841, 7.67893, 4.35171) and *ab* plane (bottom of **b**) with the slice position of (4.02830, 7.63500, 7.43644), respectively. Isovalue increases from blue to red, and the maximum electron localization function value is scaled to 4.209 × 10^−2^ for the two electron localization function diagrams

In order to achieve a deep investigation about the structure-property relationship, theoretical calculations based on density functional theory methods were performed^42–44^. The total and partial density of states projected on the constitutional atoms of *Pna*2_1_-BMBF crystal are given in Fig. 7a. Clearly, the energy bands can be divided into several regions. The uppermost part of valence bands from −7.5 to 0 eV is essentially dominated by O 2*p* states and an appreciable contribution of F 2*p* orbitals with a small amount of B 2*p* states. The obvious narrow band with sharp peak locates at about −10.7 eV entirely dominated by Ba 5*p* orbitals and also with a small contribution of O 2*p* states. The last part from −19.4 to −15.9 eV is mainly generated from O 2*s*, O 2*p*, and B 2*s*, B 2*p*, indicating strong *sp* hybridization of the B and O orbitals in the [BO_3_]^3−^ groups. While the bottom of the conduction band is essentially composed of Ba 5*d*, Ba 5*s*, B 2*p*, and O 2*p* orbitals. In principle, the optical properties are mainly determined by the electronic transitions among the states near Fermi level, which are mainly occupied by the O 2*p* nonbonding orbitals, B–O hybridization states and Ba 5*d* states in *Pna*2_1_-BMBF. Besides, the interatomic interactions within the BO_3_ units and MgO_4_F_2_ octahedra can be clearly visualized by the electron localization function diagrams. Results show the ionic character of Mg–O and Mg–F bonds in the MgO_4_F_2_ octahedra as well as the covalent nature of B–O bonds in the [BO_3_]^3−^ units (Fig. 7b). Through the Mulliken population analysis, the same conclusions can also be substantiated by the calculated overlap populations for Mg–F, Mg–O and B–O, which are 0.12–0.21, 0.21–0.55, and 0.85–0.97, respectively (higher populations reflect stronger covalence).

**Fig. 8:**
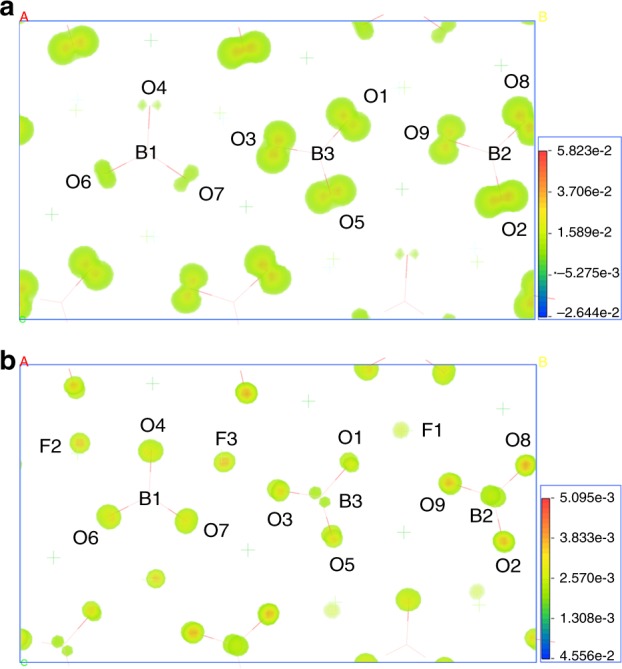
The second-harmonic generation density analysis of *Pna*2_1_-Ba_3_Mg_3_(BO_3_)_3_F_3_. **a** The density maps of the occupied orbitals in virtual electron process of the second-harmonic generation tensor *d*_33_. **b** The density maps of the unoccupied orbitals in virtual electron process of the second-harmonic generation tensor *d*_33_ of *Pna*2_1_-Ba_3_Mg_3_(BO_3_)_3_F_3_. Isovalue increases from blue to red, and the maximum electron localization function values are scaled to 5.823 × 10^−2^ for occupied orbitals (**a**) and 5.095 × 10^−2^ for unoccupied orbitals (**b**), respectively

The NLO properties of *Pna*2_1_-BMBF were also calculated using a theoretical technique under the static limit within the length gauge. The calculated SHG tensors are evaluated to be *d*_33_ = 0.47, *d*_32_ = −0.39, and *d*_31_ = 0.09 pm/V, respectively. These values are well consistent with experimental values presented above (*d*_33_ = 0.51 pm/V). The SHG density analysis can be used to intuitively and accurately display the SHG-contributed orbitals for an NLO crystal. Furthermore, we investigated the SHG origin of *Pna*2_1_-BMBF from the perspective of orbital analysis using SHG density analysis. And the SHG process is denoted by two virtual transition processes, namely virtual electron (VE) and virtual hole processes. The contributions of VE process to *d*_31_, *d*_32_, and *d*_33_ SHG tensors are up to 83.7%, 81.6%, and 86.5%, respectively, indicating that the SHG effects of three tensors mainly originate from the VE processes. Thus, only the largest *d*_33_ SHG tensor of VE process is analyzed and the results are drawn in Fig. 8. In the occupied states, the nonbonding 2*p* orbitals of O (1, 2, 3, 5, 8, 9) atoms in [B(2)O_3_]^3−^ and [B(3)O_3_]^3−^ groups give more dominant contributions to the SHG effects, while for the occupied states, *π* orbitals of the [B(1, 2, 3)O_3_]^3−^ units as well as 2*p* orbitals of F (1, 2, 3) atoms in the MgO_4_F_2_ octahedra give more dominant contributions for the SHG effects, revealing the *p*–(*p*, π*) charge-transfer excitation mechanism in *Pna*2_1_-BMBF. Both occupied and unoccupied states of the Ba cations, in analogy to alkali metal and alkali earth metal ions in other NLO crystals, have very little contribution on the SHG effect, which agrees well with the anionic group theory^10^. To sum up, the large SHG response of *Pna*2_1_-BMBF can be regarded as the synergistic effects of the planar [BO_3_]^3−^ and distorted MgO_4_F_2_ octahedra.

In summary, by starting from the SBBO structure, we designed and synthesized two beryllium-free SBBO-like borates through chemical cosubstitution strategy. A high-quality *Pna*2_1_-BMBF crystal with dimensions up to 16 × 14 × 8 mm^3^ has been grown by the top-seeded solution growth method with a BaF_2_-H_3_BO_3_-LiF flux system. Linear and nonlinear optical measurements reveal a sufficiently large SHG coefficient (*d*_33_ = 0.51 pm/V), suitable birefringence (0.045@532 nm), type I phase-matchability, and chemical stability. Remarkably, *Pna*2_1_-BMBF has a large LDT (~6.2 GW/cm^2^), a deep-UV cutoff edge (*λ* ~ 184 nm), a weak anisotropic thermal expansion as well as the capacity of insolubility in water, these optical properties could be comparable or superior to that of commercial NLO materials *β*-BBO (Supplementary Table 10). Thus, these merits on optical properties make *Pna*2_1_-BMBF a promising UV NLO material.

## Methods

### Synthesis and crystal growth

Polycrystalline samples of targeted *Pna*2_1_-BMBF were synthesized through solid-state reaction method by mixing the raw materials BaF_2_, BaCO_3_, MgO, and H_3_BO_3_ according to the molar ratio of 3:3:6:6. The mixture was preheated at 400 °C for 48 h. After that, the temperature was gradually raised to 600 °C for *Pna*2_1_-BMBF, with several intermediate mixings and grindings. Single crystals of *Pna*2_1_-BMBF were grown by top-seeded solution growth method with the flux BaF_2_-H_3_BO_3_-LiF at a molar ratio of *Pna*2_1_-BMBF/H_3_BO_3_/BaF_2_/LiF = 1:1:0.5:3. A mixture of *Pna*2_1_-BMBF polycrystalline samples, BaF_2_, H_3_BO_3_, and LiF were placed into a Φ 30 × 30 mm platinum crucible and melted at 750 °C in a temperature-programmable electric furnace. The solution was held at 750 °C for 24 h to form a homogeneous solution. After that, cooled to 715 °C and dipped the *Pna*2_1_-BMBF seed crystal into the solution and held for 2 h that allowed the seed crystal surface to melt. The solution was slowly cooled to the saturation temperature about 712 °C. Then the *Pna*2_1_-BMBF crystal was grown by cooling the solution at a rate of 0.2 °C/d to 706 °C. When the growth procedure finished, the *Pna*2_1_-BMBF crystal was drawn out of the solution and cooled down to room temperature at a rate of 20 °C/h. While for *P*$\bar 6$2*m*-BMBF single crystals, the loaded compositions are Ba(NO_3_)_2_ (0.5227 g, 2 mmol), BaF_2_ (0.3507 g, 2 mmol), MgO (0.0403 g, 1 mmol) and H_3_BO_3_ (0.4328 g, 7 mmol). All the reagents were mixed homogeneously and transferred to a platinum crucible. The samples were heated to 880 °C in 24 h and held at this temperature for 72 h and then cooled to 700 °C at a rate of 1.0 °C/h, after that, cooled to room temperature at a rate of 15 °C/h. During the process of crystallization, colorless and transparent *P*$\bar 6$2*m*-BMBF crystals were formed in the platinum crucible.

### Characterization

The selected single crystals with dimensions up to 0.12 × 0.10 × 0.01 mm^3^ for *Pna*2_1_-BMBF and 0.16 × 0.07 × 0.06 mm^3^ for *P*$\bar 6$2*m*-BMBF were glued on glass fibers for structure determination by single-crystal X-ray diffractometer. An APEX II CCD diffractometer equipped with monochromatic Mo K*α* radiation was used for the single-crystal data collection. The Bruker Suite software package was used to reduce the collected data. The numerical absorption corrections were carried out with the SADABS program and integrated with the SAINT program^45^. The original structures were established by the direct method and refined by the full-matrix least-squares program on SHELXL^46^. The PLATON program was used for checking the possible missing symmetry elements, but no higher symmetries were found^47^. Investigation of the thermal behavior *Pna*2_1_-BMBF crystal was performed using a NETZSCH STA 449 C simultaneous thermal analyzer. The sample of 6.5 mg was enclosed in a platinum crucible and heated from 40 to 1200 °C at a rate of 5 °C/min. The measurements were carried out in an atmosphere of flowing N_2_. The powder X-ray diffraction data of *Pna*2_1_-BMBF were collected at room temperature using an automated Bruker D2 X-ray diffractometer (Supplementary Figure 4). The transmittance spectra of *Pna*2_1_-BMBF water were collected by a Solid Spec-3700DUV spectrophotometer for the range of 180–2500 nm in an atmosphere of flowing N_2_ and by a Shimadzu IRAffinity-1 Fourier spectrometer for the range extended from 2500 to 4000 nm (Supplementary Figure 5). The powder SHG measurements for targeted *Pna*2_1_- and *P*$\bar 6$2*m*-BMBF were carried out on the basis of the Kurtz–Perry method^41^. Polycrystalline samples of *Pna*2_1_- and *P*$\bar 6$2*m*-BMBF were ground and sieved into the following particle size ranges: 20–38; 38–55; 55–88; 88–105; 105–150; 150–200; and 200–250 μm. During the measurement, *Pna*2_1_- and *P*$\bar 6$2*m*-BMBF samples were irradiated with a Q-switched Nd:YAG laser with light wavelength of 1064 nm. Then the intensity of the frequency-doubled of 532 nm output emitted from *Pna*2_1_- and *P*$\bar 6$2*m*-BMBF were recorded by a digital oscillometer equipped with a photomultiplier tube. The microcrystalline KDP samples with the same particle sizes were used as references. For LDT test, the crystals were cut plane parallel and polished to optical quality using a Unipol-300 grinding/polishing machine. A Q-switched Nd:YAG laser with a pulse width of 10 ns and repetition frequency of 10 kHz was employed as the fundamental light source (1064 nm). The second-harmonic signal generated from the sample wafer was detected by a side window photomultiplier tube, averaged by a fast-gated integrator and boxcar average, and then automatically recorded by a computer. A cut-KDP (110) wafer was used as a reference. The refractive index measurements of *Pna*2_1_-BMBF were carried out using (100), (010), and (001) crystal plates on the Metricon model 2010/M prism coupler (Metricon Co.) at five wavelengths (406.9, 514.0, 636, 964.8, and 1546.7 nm), and the accuracy of the measurements is estimated to be 2 × 10^−4^. Maker fringe measurements of *Pna*2_1_-BMBF were performed on two crystal wafers cut perpendicular to the *a*- and *b*-crystallographic axes in order to measure *d*_31_, *d*_32_, and *d*_33_ NLO coefficients. Then, the data were fitted according to the Maker fringe theory. The detected second-harmonic power *P*_2*ω*_ is expressed as the following Eq.(3):

3 $$P_{2\omega }\left( \theta \right) = \frac{{512\pi ^2}}{{cw^2}}d^2P_\omega ^2f\left( {n,\theta } \right)\sin ^2\Psi$$

4 $$f\left( {n,\theta } \right) = \frac{1}{{\left( {n_\omega ^2 - n_{2\omega }^2} \right)^2}}t_\omega ^4T_{2\omega }p^2\left( \theta \right)R\left( \theta \right)\beta \left( \theta \right)$$

where *c* is the speed of light in vacuum, *w* is the radius of light beam, *n*_*ω*_ and *n*_2*ω*_ are the refractive indices under the fundamental and harmonic wavelength, *d* is the NLO coefficient, *P*_*ω*_ is the fundamental beam power, Ψ = 2*πL*(*n*_*ω*_cos*θ*_*ω*_ − *n*_2*ω*_cos*θ*_2*ω*_)/*λ*_*ω*_, and sin^2^Ψ determines the minima oscillating position. *t*_*ω*_ and *t*_2*ω*_ are the transmission coefficients of fundamental wave and harmonic wave, *p*(*θ*) is the projection factor, *R*(*θ*) is the incident multiple-reflection correction, and *β*(*θ*) is the beam size correction. The *f* (*n*, *θ*) part depicts the envelope of the Maker fringe. By fitting the calculated and measured Maker fringes, a constant, *C* *=* 512*πd*^2^*P*^2^_*ω*_/(*cω*^2^) can be obtained. The magnitude of NLO coefficients of *Pna*2_1_-Ba_3_Mg_3_(BO_3_)_3_F_3_ can be determined relatively to *d*_36_ of KDP crystal, and the coefficient equation can be expressed as:

5 $$d_{{\mathrm{sample}}} = \sqrt {\frac{{C_{{\mathrm{sample}}}}}{{C_{\mathrm{KDP}}}}} d_{36}\left( {{\mathrm{KDP}}} \right)$$

### Computational methods

First-principles calculations of *Pna*2_1_-BMBF based on density functional theory were performed by a planewave pseudopotential calculation package CASTEP^48^. The exchange and correlation effects were treated by Perdew–Burke–Ernzerhof in the generalized gradient approximation^49^. The interactions between the ionic cores and electrons were described by norm-conserving pseudopotentials^50^. The cutoff energy for the planewave basis was 990 eV and the Brillouin zone was sampled by 4 × 2 × 3 Monkhorst-Pack *k*-point.

### Data availability

The authors declare that the data supporting the findings of this study are available within the article and Supplementary Information files, or from the corresponding authors upon reasonable request.

## Electronic supplementary material

Supplementary Information
