# Supplementary material for: Ba3Mg3(BO3)3F3 polymorphs with reversible phase transition and high performances as ultraviolet nonlinear optical materials
Source: Nat Commun. 2018 Aug 6;9:3089. doi: 10.1038/s41467-018-05575-w (PMC6078997; doi:10.1038/s41467-018-05575-w)
Supplement: Supplementary file 1 — Supplementary Information [file 41467_2018_5575_MOESM1_ESM.pdf]

Supplementary Information

$Ba_3Mg_3(BO_3)_3F_3$ polymorphs with reversible phase transition and high performances as ultraviolet nonlinear optical materials

Miriding Mutailipu, Min Zhang, Hongping Wu, Zhihua Yang, Yihan Shen,
Junliang Sun* and Shilie Pan*

Supplementary Figure 1. (**a**) The ${}^{2}_{\infty}[Mg_3O_2F_3(BO_3)_2]$ layer extending in the *bc* plane in *Pna*2$_1$-$Ba_3Mg_3(BO_3)_3F_3$. (**b**) The ${}^{2}_{\infty}[Mg_3O_2F_3(BO_3)_2]$ layer extending in the *ab* plane in $P\bar{6}2m$-$Ba_3Mg_3(BO_3)_3F_3$. $Ba_3Mg_3(BO_3)_3F_3$ series possess a similar layered crystal structures, their crystal structures are both composed of ${}^{2}_{\infty}[Mg_3O_2F_3(BO_3)_2]$ layers along the *a* axis for *Pna*2$_1$- $Ba_3Mg_3(BO_3)_3F_3$ and *c* axis for $P\bar{6}2m$-$Ba_3Mg_3(BO_3)_3F_3$, and those single layers are further connected by the Mg-F bonds to construct a three dimensional framework with tunnels running along the *c* (or *a*) axis.

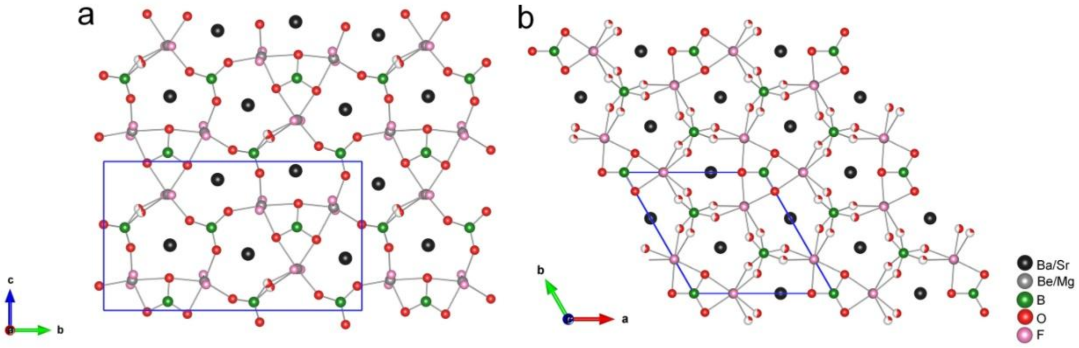

Supplementary Figure 2. The six-membered ring cluster $Mg_3O_9F_6$ in *Pna*2_1-$Ba_3Mg_3(BO_3)_3F_3$. The Mg atoms in both polymorphs are six-coordinated into the MgO_4F_2 octahedra. Three MgO_4F_2 units share three O atoms to generate a six-membered ring cluster $Mg_3O_9F_6$.

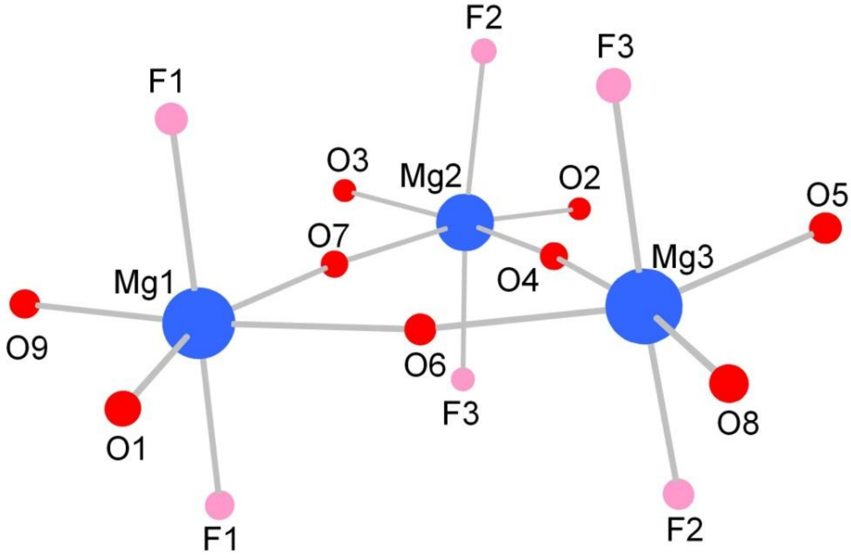

Supplementary Figure 3. The isolated $^{3}_{\infty}[Mg_3O_9F_3]$ triangular prism in *Pna*2_1-$Ba_3Mg_3(BO_3)_3F_3$. Those six-membered ring $Mg_3O_9F_6$ clusters link together via axial Mg-F bonds to build an isolated $^{3}_{\infty}[Mg_3O_9F_3]$ triangular prism.

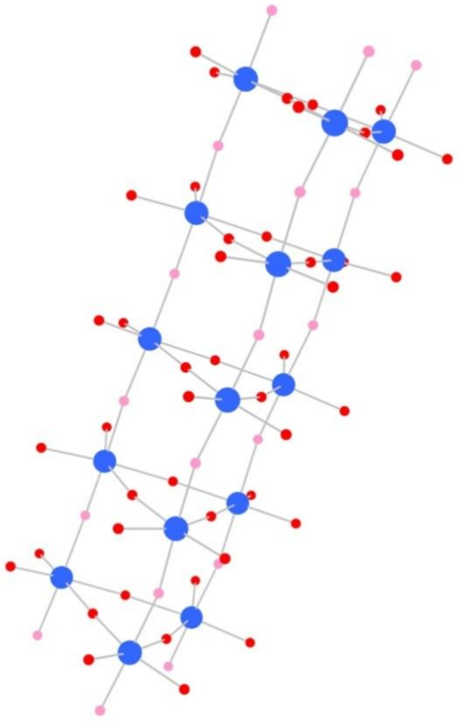

Supplementary Figure 4. (**a**) The calculated X-ray diffraction patterns of *Pna*2_1- and $P\bar{6}2m$-$Ba_3Mg_3(BO_3)_3F_3$. (**b**) The experimental (red line) and calculated (black line) X-ray diffraction patterns of *Pna*2_1-$Ba_3Mg_3(BO_3)_3F_3$. The peaks marked by blue arrows mean the difference between the X-ray diffraction patterns of two polymorphs, which can help us to easily observe the phase transition behavior by analyzing the collected high-temperature *in situ* powder X-ray diffraction data. Polycrystalline samples of *Pna*2_1-$Ba_3Mg_3(BO_3)_3F_3$ were synthesized by the solid-state reaction method. The purity of as-prepared polycrystalline samples is confirmed by the powder X-ray diffraction patterns.

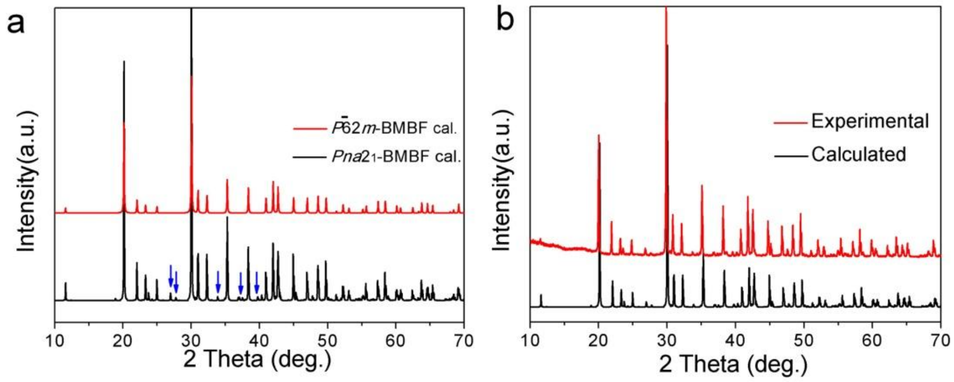

Supplementary Figure 5. The infrared spectrum of *Pna*2_1-$Ba_3Mg_3(BO_3)_3F_3$. The infrared spectrum of *Pna*2_1-$Ba_3Mg_3(BO_3)_3F_3$ is plotted in Supplementary Figure 5. The sharp absorption peak at about 1269 cm^{-1} is mainly attributed to the asymmetric stretching vibrations of BO_3, and symmetric stretching vibrations of BO_3 locate around 750 and 711 cm^{-1}.

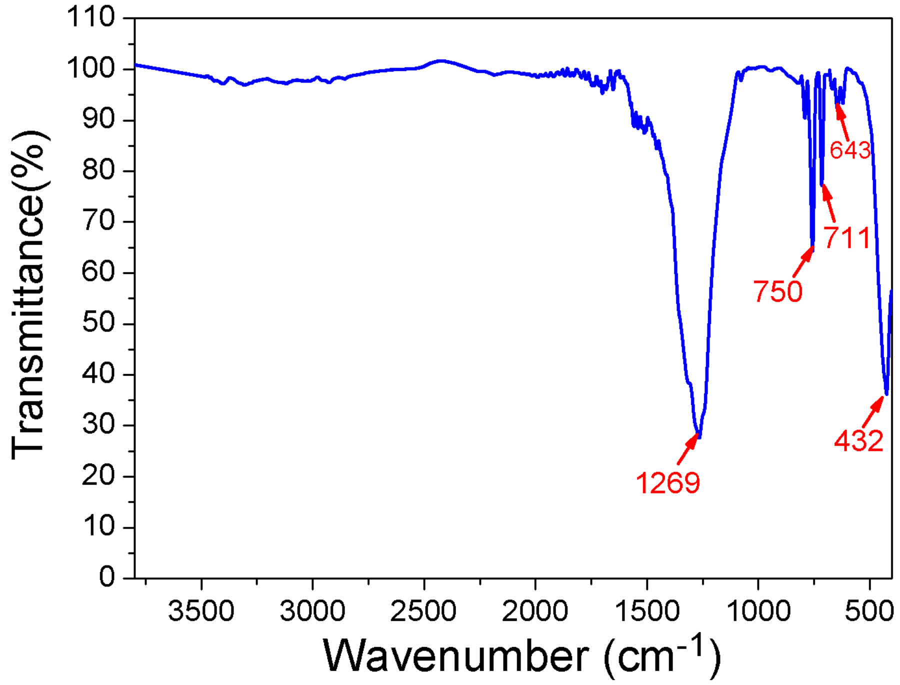

Supplementary Table 1. Crystallographic data for $Pna2_1$-and $P\bar{6}2m$-$Ba_3Mg_3(BO_3)_3F_3$

empirical formula	$Ba_3Mg_3(BO_3)_3F_3$	$Ba_3Mg_3(BO_3)_3F_3$
formula weight	718.38	718.38
crystal system	Orthorhombic	Hexagonal
space group	$Pna2_1$ (No. 33)	$P\bar{6}2m$ (No. 189)
a (Å)	8.0740(3)	8.804(3)
b (Å)	15.3072(7)	8.804(3)
c (Å)	8.8218(4)	4.025(3)
Z	4	1
volume (Å^3)	1090.29(8)	270.2(2)
density (g/cm^3)	4.376	4.415
abs coeff (mm^{-1})	10.974	11.071
F(000)	1272	318
cryst size (mm^3)	0.12 × 0.10 × 0.01	0.16 × 0.07 × 0.06
the range for data collection (deg)	3.67 to 27.09	2.67 to 27.32
index ranges	$-10 \le h \le 10$, $-19 \le k \le 19$, $-11 \le l \le 11$	$-11 \le h \le 11$, $-11 \le k \le 11$, $-2 \le l \le 5$
reflns collected/unique	11605 / 2377 [R(int) = 0.0243]	1695 / 261 [R(int) = 0.0386]
completeness (%)	99.8	100
data/restraints/param	2377 / 1 / 195	261 / 0 / 32
Goodness-of-fit on F^2	1.233	1.251
final R indices $[F_o^2>2\sigma(F_o^2)]^a$	R_1 = 0.0118, wR_2 = 0.0249	R_1 = 0.0204, wR_2 = 0.0394
R indices (all data) ^{a}	R_1 = 0.0120, wR_2 = 0.0250	R_1 = 0.0207, wR_2 = 0.0395
absolute structure parameter	0.000(10)	-0.07(6)
extinction coeff.	0.00063(4)	0.0248(18)
largest diff peak and hole (e/Å^3)	0.366 and -0.438	0.589 and -0.936

R = Residual factor

$^aR_1 = \Sigma||F_o| - |F_c||/\Sigma|F_o|$ and $wR_2 = [\Sigma w(F_o^2 - F_c^2)^2/ \Sigma wF_o^4]^{1/2}$ for $F_o^2 > 2\sigma(F_o^2)$.

Supplementary Table 2. Atomic coordinates equivalent isotropic displacement parameters for *Pna*2_1-$Ba_3Mg_3(BO_3)_3F_3$. U_{eq} is defined as one-third of the trace of the orthogonalized U_{ij} tensor.

Atoms	*x*	*y*	*z*	U_{eq}(Å^2)
Ba(1)	-0.0102(1)	0.0589(1)	0.8789(1)	0.007(1)
Ba(2)	-0.0256(1)	0.2432(1)	0.4379(1)	0.007(1)
Ba(3)	-0.5114(1)	0.0640(1)	0.8499(1)	0.007(1)
Mg(1)	0.2298(1)	0.2485(1)	0.7796(2)	0.006(1)
Mg(2)	-0.2759(1)	0.1098(1)	1.1932(1)	0.006(1)
Mg(3)	0.2309(1)	0.1104(1)	0.2037(1)	0.007(1)
B(1)	0.2326(4)	0.2483(2)	1.0597(5)	0.007(1)
B(2)	-0.2599(4)	0.0825(3)	0.5560(4)	0.006(1)
B(3)	0.2454(4)	0.0815(2)	0.5561(4)	0.006(1)
O(1)	0.2779(15)	0.1450(8)	0.6566(13)	0.013(1)
O(1')	0.2273(11)	0.1343(6)	0.6826(9)	0.013(1)
O(2)	-0.2429(2)	0.0632(1)	0.4047(3)	0.009(1)
O(3)	-0.3122(3)	0.0005(1)	1.0773(2)	0.014(1)
O(4)	0.2292(3)	0.2536(1)	0.2176(3)	0.008(1)
O(5)	0.1753(2)	0.1042(1)	0.4215(2)	0.013(1)
O(6)	0.2436(2)	0.1680(1)	0.9896(2)	0.008(1)
O(7)	0.2214(2)	0.3241(1)	0.9751(2)	0.007(1)
O(8)	-0.2427(2)	0.0178(2)	0.6641(2)	0.011(1)
O(9)	-0.2906(2)	0.1673(1)	0.6032(2)	0.010(1)
F(1)	-0.0205(2)	0.2374(1)	0.7749(2)	0.019(1)
F(2)	-0.0239(2)	0.3859(1)	0.2402(2)	0.012(1)
F(3)	-0.0241(2)	0.1036(1)	1.1732(2)	0.016(1)

Supplementary Table 3. Anisotropic displacement parameters (Å^2) for *Pna*2_1-$Ba_3Mg_3(BO_3)_3F_3$

**Atom**	U_{11}	U_{22}	U_{33}	U_{23}	U_{13}	U_{12}
Ba(1)	0.007(1)	0.006(1)	0.008(1)	0.000(1)	0.000(1)	-0.001(1)
Ba(2)	0.006(1)	0.009(1)	0.007(1)	0.000(1)	0.000(1)	-0.001(1)
Ba(3)	0.007(1)	0.007(1)	0.007(1)	-0.001(1)	-0.002(1)	0.000(1)
Mg(1)	0.009(1)	0.004(1)	0.006(1)	-0.001(1)	-0.002(1)	0.001(1)
Mg(2)	0.007(1)	0.006(1)	0.006(1)	0.000(1)	-0.001(1)	0.000(1)
Mg(3)	0.009(1)	0.006(1)	0.005(1)	0.000(1)	-0.001(1)	-0.001(1)
B(1)	0.006(2)	0.007(2)	0.007(2)	-0.002(1)	-0.001(1)	0.000(1)
B(2)	0.004(1)	0.005(2)	0.010(2)	0.000(1)	0.000(1)	-0.001(1)
B(3)	0.006(1)	0.005(2)	0.006(1)	0.000(1)	0.001(1)	-0.003(1)
O(1)	0.027(4)	0.007(2)	0.004(3)	-0.002(2)	0.003(2)	0.004(3)
O(1')	0.027(4)	0.007(2)	0.004(3)	-0.002(2)	0.003(2)	0.004(3)
O(2)	0.012(1)	0.009(1)	0.006(1)	0.001(1)	0.001(1)	0.003(1)
O(3)	0.025(1)	0.006(1)	0.010(1)	-0.002(1)	-0.002(1)	0.005(1)
O(4)	0.010(1)	0.008(1)	0.006(1)	0.001(1)	-0.001(1)	0.000(1)
O(5)	0.018(1)	0.013(1)	0.007(1)	-0.001(1)	-0.003(1)	0.005(1)
O(6)	0.011(1)	0.007(1)	0.007(1)	-0.001(1)	-0.001(1)	0.000(1)
O(7)	0.010(1)	0.005(1)	0.005(1)	0.000(1)	0.000(1)	0.001(1)
O(8)	0.016(1)	0.010(1)	0.006(1)	0.003(1)	-0.001(1)	-0.002(1)
O(9)	0.012(1)	0.008(1)	0.009(1)	-0.001(1)	-0.002(1)	0.002(1)
F(1)	0.009(1)	0.033(1)	0.015(1)	0.006(1)	-0.003(1)	-0.010(1)
F(2)	0.009(1)	0.010(1)	0.018(1)	0.002(1)	-0.002(1)	0.000(1)
F(3)	0.007(1)	0.031(1)	0.010(1)	0.003(1)	-0.001(1)	0.001(1)

Supplementary Table 4. Selected bond lengths (Å) for *Pna*2_1-$Ba_3Mg_3(BO_3)_3F_3$

**Bond**	**Length**	**Bond**	**Length**
Ba(1)-F(3)	2.6870(18)	Ba(3)-F(1)$^{\#2}$	3.112(2)
Ba(1)-O(8)	2.741(2)	Mg(1)-O(1)	1.960(12)
Ba(1)-O(2)$^{\#1}$	2.7781(18)	Mg(1)-O(9)$^{\#4}$	2.026(2)
Ba(1)-O(6)	2.819(2)	Mg(1)-F(1)$^{\#4}$	2.028(2)
Ba(1)-O(1')	2.830(9)	Mg(1)-F(1)	2.029(2)
Ba(1)-O(5)$^{\#1}$	2.8543(18)	Mg(1)-O(7)	2.078(2)
Ba(1)-F(1)	2.884(2)	Mg(1)-O(6)	2.228(3)
Ba(1)-O(7)$^{\#2}$	2.9372(19)	Mg(2)-O(3)	1.983(2)
Ba(1)-F(3)$^{\#3}$	3.0913(19)	Mg(2)-O(2)$^{\#10}$	2.015(2)
Ba(1)-O(3)	3.132(2)	Mg(2)-F(3)	2.0432(18)
Ba(1)-O(1)	3.316(11)	Mg(2)-F(2)$^{\#11}$	2.0456(18)
Ba(2)-O(5)	2.6800(17)	Mg(2)-O(4)$^{\#11}$	2.103(2)
Ba(2)-O(9)$^{\#4}$	2.757(2)	Mg(2)-O(7)$^{\#2}$	2.174(2)
Ba(2)-O(4)$^{\#2}$	2.774(2)	Mg(3)-O(5)	1.976(2)
Ba(2)-F(2)	2.7942(18)	Mg(3)-O(8)$^{\#3}$	1.995(2)
Ba(2)-O(4)	2.834(2)	Mg(3)-F(2)$^{\#4}$	2.0067(19)
Ba(2)-O(9)	2.838(2)	Mg(3)-F(3)$^{\#5}$	2.0790(18)
Ba(2)-F(1)	2.975(2)	Mg(3)-O(6)$^{\#5}$	2.087(2)
Ba(2)-O(1)$^{\#2}$	3.028(11)	Mg(3)-O(4)	2.195(2)
Ba(2)-F(3)$^{\#5}$	3.1653(19)	B(1)-O(6)	1.378(4)
Ba(3)-O(1)$^{\#6}$	2.709(13)	B(1)-O(7)	1.382(4)
Ba(3)-O(3)	2.749(2)	B(1)-O(4)$^{\#11}$	1.395(5)
Ba(3)-O(7)$^{\#2}$	2.7730(19)	B(2)-O(2)	1.374(4)
Ba(3)-O(1')$^{\#6}$	2.791(9)	B(2)-O(8)	1.382(4)
Ba(3)-O(8)	2.810(2)	B(2)-O(9)	1.385(4)
Ba(3)-O(2)$^{\#7}$	2.8215(19)	B(3)-O(1)	1.341(13)
Ba(3)-O(6)$^{\#6}$	2.823(2)	B(3)-O(5)	1.361(4)
Ba(3)-F(2)$^{\#8}$	2.9072(18)	B(3)-O(3)$^{\#3}$	1.378(4)
Ba(3)-O(3)$^{\#9}$	2.964(2)		

Symmetry transformations used to generate equivalent atom:

#1 -x, -y, z+1/2; **#2** x-1/2, -y+1/2, z; **#3** -x, -y, z-1/2 ;
#4 x+1/2, -y+1/2, z; **#5** x, y, z-1; **#6** x-1, y, z;
#7 -x-1, -y, z+1/2; **#8** -x-1/2, y-1/2, z+1/2; **#9** -x-1, -y, z-1/2;
#10 x, y, z+1; **#11** x-1/2, -y+1/2, z+1.

Supplementary Table 5. Atomic coordinates equivalent isotropic displacement parameters for $P\bar{6}2m$-$Ba_3Mg_3(BO_3)_3F_3$. U_{eq} is defined as one-third of the trace of the orthogonalized U_{ij} tensor.

Atoms	*x*	*y*	*z*	U_{eq}(Å^2)
Ba(1)	0.6206(1)	1.0000	0.5000	0.024(1)
Mg(1)	1.0000	1.2802(4)	0.0000	0.021(1)
B(1)	0.3333	0.6667	0.0000	0.012(3)
B(2)	1.0000	1.0000	0.0000	0.021(5)
F(1)	1.0000	1.2842(8)	-0.5000	0.065(2)
O(1A)	0.4655(15)	0.6298(19)	0.0000	0.031(3)
O(1B)	0.5000(20)	0.7240(20)	-0.0970(50)	0.031(3)
O(2)	1.1569(8)	1.1569(8)	0.0000	0.024(2)

Supplementary Table 6. Anisotropic displacement parameters (Å^2) for $P\bar{6}2m$-$Ba_3Mg_3(BO_3)_3F_3$

**Atom**	U_{11}	U_{22}	U_{33}	U_{23}	U_{13}	U_{12}
Ba(1)	0.018(1)	0.029(1)	0.030(1)	0.000	0.000	0.015(1)
Mg(1)	0.011(2)	0.010(1)	0.043(3)	0.000	0.000	0.006(1)
B(1)	0.013(4)	0.013(4)	0.008(7)	0.000	0.000	0.007(2)
B(2)	0.007(6)	0.007(6)	0.050(15)	0.000	0.000	0.003(3)
F(1)	0.110(7)	0.047(3)	0.060(6)	0.000	0.000	0.055(4)
O(1A)	0.012(4)	0.020(5)	0.066(9)	0.000	0.000	0.013(5)
O(1B)	0.012(4)	0.020(5)	0.066(9)	0.000	0.000	0.013(5)
O(2)	0.013(3)	0.013(3)	0.044(5)	0.000	0.000	0.005(3)

Supplementary Table 7. Selected bond lengths (Å) for $P\bar{6}2m$-$Ba_3Mg_3(BO_3)_3F_3$

**Bond Length**		**Bond Length**	
Ba(1)-O(1B)$^{\#1}$	2.660(19)	Mg(1)-O(1B)$^{\#14}$	1.993(14)
Ba(1)-O(1B)$^{\#2}$	2.660(19)	Mg(1)-O(1B)$^{\#4}$	1.993(14)
Ba(1)-O(1B)$^{\#3}$	2.660(19)	Mg(1)-F(1)	2.0127(13)
Ba(1)-O(1B)$^{\#4}$	2.660(19)	Mg(1)-F(1)$^{\#2}$	2.0127(13)
Ba(1)-O(1A)$^{\#5}$	2.795(10)	B(1)-O(1B)$^{\#1}$	1.349(15)
Ba(1)-O(1A)$^{\#6}$	2.795(10)	B(1)-O(1B)$^{\#15}$	1.349(15)
Ba(1)-O(1A)$^{\#7}$	2.795(10)	B(1)-O(1B)$^{\#16}$	1.349(15)
Ba(1)-O(1A)$^{\#8}$	2.795(10)	B(1)-O(1B)	1.349(15)
Ba(1)-O(2)$^{\#9}$	2.809(5)	B(1)-O(1B)$^{\#17}$	1.349(15)
Ba(1)-O(2)$^{\#10}$	2.809(5)	B(1)-O(1B)$^{\#7}$	1.349(15)
Ba(1)-F(1)$^{\#11}$	2.962(7)	B(1)-O(1A)$^{\#7}$	1.355(9)
Ba(1)-F(1)$^{\#2}$	3.010(2)	B(1)-O(1A)$^{\#15}$	1.355(9)
Mg(1)-O(1A)$^{\#12}$	1.967(11)	B(1)-O(1A)	1.355(9)
Mg(1)-O(1A)$^{\#4}$	1.967(11)	B(2)-O(2)$^{\#12}$	1.381(7)
Mg(1)-O(1B)$^{\#13}$	1.993(14)	B(2)-O(2)	1.381(7)
Mg(1)-O(1B)$^{\#12}$	1.993(14)	B(2)-O(2)$^{\#10}$	1.381(7)

Symmetry transformations used to generate equivalent atom:
# 1 x, y, -z; **# 2** x, y, z+1; **# 3** x-y+1, -y+2, z+1;
# 4 x-y+1, -y+2, -z; **# 5** -x+1, -x+y+1, -z+1; **# 6** -x+1, -x+y+1, -z;
# 7 -y+1, x-y+1, z; **# 8** -y+1, x-y+1, z+1; **# 9** -y+2, x-y+1, z+1;
# 10 -y+2, x-y+1, z; **# 11** -x+y, -x+2, z+1; **# 12** -x+y+1, -x+2, z;
# 13 -x+y+1, -x+2, -z; **# 14** x-y+1, -y+2, z; **# 15** -x+y, -x+1, z;
# 16 -x+y, -x+1, -z; **# 17** -y+1, x-y+1, -z.

Supplementary Table 8. The interlayer bonding and calculated electrostatics force in KBBF and $Ba_3Mg_3(BO_3)_3F_3$ series.

Crystals	Bonds	q_1	q_2	Lengths	|F|	×KBBF
KBBF	K-F	1	1	2.757	0.13	1.0
$Pna2_1$-$Ba_3Mg_3(BO_3)_3F_3$	Mg1-F	2	1	2.029	0.49	3.8
	Mg1-F	2	1	2.028	0.49	3.8
	Mg2-F	2	1	2.043	0.48	3.7
	Mg2-F	2	1	2.045	0.48	3.7
	Mg3-F	2	1	2.007	0.48	3.7
	Mg3-F	2	1	2.079	0.46	3.5
$P\bar{6}2m$-$Ba_3Mg_3(BO_3)_3F_3$	Mg1-F	2	1	2.013	0.49	3.8
	Mg1-F	2	2	2.013	0.49	3.8

Supplementary Table 9. Experimental and calculated refractive indices for *Pna*2_1-$Ba_3Mg_3(BO_3)_3F_3$

**λ/μm**	$\boldsymbol{n_x}$		$\boldsymbol{n_y}$		$\boldsymbol{n_z}$		**Δ*n***
	**Exp.**	**Cal.**	**Exp.**	**Cal.**	**Exp.**	**Cal.**	
0.4069	1.6406	1.6406	1.6837	1.6838	1.6882	1.6882	0.0476
0.5140	1.6278	1.6277	1.6712	1.6708	1.6737	1.6733	0.0459
0.6360	1.6199	1.6201	1.6621	1.6626	1.6642	1.6647	0.0443
0.9468	1.6112	1.6111	1.6532	1.6530	1.6553	1.6550	0.0441
1.5467	1.6031	1.6031	1.6463	1.6463	1.6475	1.6475	0.0444

Supplementary Table 10. Properties for β-BaB_2O_4 and $Pna2_1$-$Ba_3Mg_3(BO_3)_3F_3$

**Properties**	***Pna*2₁-Ba₃Mg₃(BO₃)₃F₃**	***β*-BaB₂O₄**
λ_{cutoff} (nm)	184	189
Laser damage threshold (GW/cm^2)	6.2 (1064 nm, 10 ns, 10 Hz)	6.0 (1064 nm, 10 ns, 10 Hz)
Thermal expansion coefficients (K^{-1})	$\alpha_a = 2.17 \times 10^{-5}$<br>$\alpha_b = 1.63 \times 10^{-5}$<br>$\alpha_c = 1.53 \times 10^{-5}$<br>$\alpha_a/\alpha_c = 1.43$	$\alpha_a = 4 \times 10^{-6}$<br>$\alpha_b = 4 \times 10^{-6}$<br>$\alpha_c = 36 \times 10^{-6}$<br>$\alpha_c/\alpha_a = 9$
Birefringence @532 nm	0.045	0.1195
Solubility in water	insolubility	dissolution
SHG coefficients (pm/V)	$d_{33} = 0.51$	$d_{11} = 1.60$
Shortest phase matching wavelength (nm) for SHG	310	205

Supplementary Discussion

To further study the thermodynamic processes of *Pna*2$_1$-$Ba_3Mg_3(BO_3)_3F_3$, thermal gravimetric analysis TGA and differential scanning calorimetry DSC curves were obtained on the heating as well as cooling cycles. Obviously, there are two remarkable endothermic peaks (741.8 and 1108.9 ℃) on the heating curve and two exothermic peaks (634.1 and 1035.1 ℃) on the cooling curve, respectively. In order to confirm the ascription of these peaks, high-temperature *in situ* powder X-ray diffraction experiments combined with calcinations tests were performed. Results show that *Pna*2$_1$-$Ba_3Mg_3(BO_3)_3F_3$ is stable up to 400 ℃ and then transfers into a high temperature phase $P\bar{6}2m$-$Ba_3Mg_3(BO_3)_3F_3$. When the temperature approaches to 740 ℃, the high temperature phase starts to decompose into $Ba_2Mg(BO_3)_2$ (PDF no. 82-1883). Ulteriorly, circular heating and cooling in the region of 100-650 ℃ can confirm the reversible phase transition behaviors, *Pna*2$_1$- $Ba_3Mg_3(BO_3)_3F_3$ → $P\bar{6}2m$-$Ba_3Mg_3(BO_3)_3F_3$ → *Pna*2$_1$-$Ba_3Mg_3(BO_3)_3F_3$. Stated thus, the thermal behavior of $Ba_3Mg_3(BO_3)_3F_3$ is clear, the first endothermic peak (741.8 ℃) on the heating curve is attributed to the decomposition of the high temperature phase $P\bar{6}2m$-$Ba_3Mg_3(BO_3)_3F_3$ into $Ba_2Mg(BO_3)_2$, the second one should be corresponding to the melt of the residues and $Ba_2Mg(BO_3)_2$. While for the exothermic peaks on the cooling curve, it might be regarded as the crystallization of $Ba_2Mg(BO_3)_2$ and residues. Accordingly, we know that $Ba_3Mg_3(BO_3)_3F_3$ melts incongruently and its synthesis temperature cannot be higher than 740 ℃ on the process of solid-state reaction and also appropriate flux should be introduced during the crystal growth process.
